# Effects of Aqueous Solubility and Geochemistry on CO_2_ Injection for Shale Gas Reservoirs

**DOI:** 10.1038/s41598-020-59131-y

**Published:** 2020-02-07

**Authors:** Ji Ho Lee, Jinhyung Cho, Kun Sang Lee

**Affiliations:** 1 Korea National Oil Corporation, 305, Jongga-ro, Jung-gu, Ulsan, 44538 Republic of Korea; 2 Department of Earth Resources and Environmental Engineering, Hanyang University, Seoul, 04763 Republic of Korea

**Keywords:** Natural gas, Carbon capture and storage

## Abstract

In shale gas reservoirs, CH_4_ and CO_2_ have finite aqueous solubilities at high-pressure conditions and their dissolutions in water affect the determination of the original gas in place and the CO_2_ sequestration. In addition, the dissolution of CO_2_ decreases the pH of connate water, and the geochemical reactions may thus occur in carbonate-rich shale reservoirs. The comprehensive simulations of this work quantify the effects of aqueous solubility and geochemistry on the performance CO_2_ huff-n-puff process in shale gas reservoir. Accounting for the aqueous solubility of CH_4_ increases the initial natural gas storage and natural gas production. The effect of the aqueous solubility of CO_2_ enables to sequester additional CO_2_ via solubility trapping. Considering the geochemical reactions, the application of the CO_2_ huff-n-puff process causes the dissolution of carbonate minerals and increases the porosity enhancing the gas flow and the gas recovery. Incorporation of geochemistry also predicts the less CO_2_ sequestration capacity. Therefore, this study recommends the consideration of aqueous solubility and geochemical reactions for the accurate prediction of gas recovery and CO_2_ sequestration in shale gas reservoirs during the CO_2_ huff-n-puff process.

## Introduction

Significant shale gas is reported to be distributed in the U.S. as well as Canada^1–4^. The Energy Information Administration reported that shale gas resources in U.S. are estimated to be 308 trillion cubic feet^5^. The gas production from shale gas and tight oil reservoirs is approximately half of the total gas production in the U.S. The U.S. dry natural gas production exceeded the U.S. natural gas consumption in 2017. In the shale formations, the methane occupies more than 94% of the total gas and it is stored via a number of forms such as adsorption, free, and dissolution within the pore structure^6–8^. The development of horizontal wells and multistage fracturing technologies allows the rapid expansion of natural gas production from shale reservoirs, but the production is primarily attributed to free gas obtained through the fracture network which connects separated fractures. The shale reservoirs contain organic matter and a fraction of the initial gas storage is attributed to the adsorption of CH_4_ on the internal surface areas of organic matter. The physical adsorption on both the organic matter and clays contributes to a fraction of initial natural gas storage^6,9^. However, natural depletion process with the use of the technologies (horizontal wells and multistage fracturing) hardly recovers the adsorbed gas from shale reservoirs. The rapid decline in gas production within a few years is indicative of the low production of the adsorbed shale gas via natural depletion.

The development of enhanced gas recovery (EGR) methodologies is required to overcome the low production of natural depletion process and to produce a remained CH_4_ via the adsorption mechanism from shale formations. The CO_2_ injection has been proposed as a promising EGR technology to accelerate the CH_4_ desorption by encouraging the CO_2_ adsorption in the organic-rich shale system. While the adsorptions of CH_4_ and CO_2_ depend on the conditions of shale formation, CO_2_ has a higher potential of adsorption than CH_4_. Considering the competitive adsorption behaviors of CO_2_ and CH_4_, extensive studies have investigated the EGR of CO_2_ injection in shale gas formations^10–15^. In addition, shale gas formation is proposed as a potential option for sequestrating CO_2_^16^. The CO_2_ injection is an attractive EGR incorporating CO_2_ storage in shale gas formations^10,16–19^. Experimentally, a number of factors (adsorption behaviors of CH_4_ and CO_2_, pore size distribution, geochemical reactions, fracturing, and wettability) have been evaluated for storing CO_2_ in shale formations^20–24^.

Extensive studies thoroughly investigated the potentials of EGR and CO_2_ sequestration via CO_2_ injection for shale gas reservoirs. Many studies^10–15,17,19–29^ numerically demonstrated the potentials in shale formations. However, they ignored some factors of considerable importance affecting the storage and transport of CO_2_ and CH_4_ during the CO_2_ injection into the shale gas formation. The studies^10–15,17,19–29^ neglected the solubilities of CH_4_ and CO_2_ in the formation water, which affect the gas storage of CH_4_ and CO_2_ in shale gas reservoirs. In addition, the effect of the geochemical reactions on the EGR and CO_2_ sequestration has not been investigated in the numerical studies^10–15,17,19–29^. Experimental studies^22,30–32^ have explored the role of geochemical reactions in shale formations. The study^22^ designed the characterization method consisting of optical microscope, X-ray diffraction, element analysis, low-pressure gas adsorption, and Fourier transform infrared spectroscopy to access geochemical changes when CO_2_ was injected into carbonate-rich shale rock samples. It was observed that CO_2_ dissolution in brine decreased the pH of brine resulting in mineral reactions and pore structure change. Another study^32^ also observed carbonate mineral dissolution in the system of carbonate-rich shale/formation water/CO_2_ by *in-situ* Fourier transform infrared spectroscopy. The experimental studies clarified the geochemical reactions in the system of carbonate-rich shale/formation water/CO_2_ and observed the changes in physical properties of shale rock samples. However, the experimental studies have not quantified the effect of geochemical reactions on EGR and CO_2_ storage during CO_2_ injection for shale gas reservoirs.

Therefore, it is necessary to advance the understanding of CO_2_ injection in tight shale reservoirs by implementing potential factors into the numerical simulations: (1) aqueous solubilities of CO_2_ and CH_4_ and (2) chemical reactions occurred in the system of gas/brine/shale formation. In this numerical study, an improved model of the CO_2_ injection has been developed to account for the solubilities of CO_2_ and CH_4_ in formation water and geochemical reactions in the shale gas reservoirs. The effects of potential factors on the EGR and CO_2_ storage have been analyzed with a series of runs with the developed model. The developed model is examined in the Eagle Ford shale gas reservoirs which have a high fraction of carbonate minerals. The Eagle Ford shale play is late Cretaceous in age and is located in the South Texas, USA^33,34^. It is 50 miles wide and 400 miles long and covers the 23 counties in South-Central Texas. Its depth varies between 2,500 ft and 14,000 ft and thickness ranges from 120 ft to 350 ft. The Eagle Ford shale consists of Cretaceous mudstone and carbonate that are the source rock for the Austin Chalk formation. The carbonate content of the Eagle Ford shale varies. According to Hsu and Nelson^33^, an average carbonate content of 10% was reported based on 119 samples. The EIA^34^ reported 40–90% carbonate minerals content in the Eagle Ford formation. For the carbonate-rich shale formation, this study simulates the improved model of the CO_2_ injection and investigates the roles of the potential factors on the EGR and CO_2_ sequestration of CO_2_ injection.

## Mathematical Formulations

### Adsorption

The multi-component adsorption from the gas phase on the reservoir rocks could be modeled based on an extended Langmuir isotherm model. The extended Langmuir isothermal model of CO_2_ and CH_4_ is a function of pressure and is described in Eq. 1.

1 $${\omega }_{k}=\frac{{\omega }_{k,{\rm{\max }}}{B}_{k}\,{y}_{k,g}p}{1+p\sum _{i}{B}_{i}{y}_{i,g}}$$

where *k* and *i* indicate the component, e.g., CO_2_ and CH_4_; *ω*_*k*_ denotes the moles of the adsorbed component *k* per unit mass of rock; *ω*_*k*, max_ is the maximum number of moles of adsorbed component *k* per unit mass of rock; *p* is the pressure; *y*_*i*, *g*_ is the molar fraction of the adsorbed component *k* in the gaseous phase; and *B* is a parameter of extended Langmuir isotherm model.

### Solubility in water

The gaseous components (CO_2_ and CH_4_) could dissolve in water at the high pressure condition. In shale gas reservoirs, a fraction of CH_4_ might dissolves in formation water. Once the CO_2_ is injected into the water-bearing gas reservoir, the CO_2_ dissolves in the water as well. In this system, the aqueous solubilities of CO_2_ and CH_4_ are determined by equating their fugacities in the aqueous and gaseous phases (Eq. 2). While the Peng-Robinson equation of state (PR-EoS) determines the fugacities of gaseous components in the gaseous phase, Henry’s law calculates their fugacities in the aqueous phase (Eq. 3). Introducing the Henry’s law constant, it determines the amount of dissolution in the aqueous phase at specific temperature and pressure conditions (Eq. 4). Harvey^35^ published the correlations of Henry’s law constants for CO_2_ and CH_4_ (Eqs. 5 and 6). In addition, solubilities of CO_2_ and CH_4_ in aqueous phase are affected by the aqueous salinity. The relationship between solubility and salinity is formulated by introducing a salting-out coefficient (Eq. 7). The Henry’s law constants of CO_2_ and CH_4_ at specific salinity are determined by incorporating their Henry’s law constants in pure water and salting-out coefficients. The correlations predicting the salting-out coefficients of CO_2_ and CH_4_ are developed as shown in Eqs. 8 and 9^36^.

2 $${f}_{i,g}={f}_{i,aq}$$

3 $${f}_{i,aq}={H}_{i}{x}_{i}$$

4 $$\mathrm{ln}\,{H}_{i}=\,\mathrm{ln}\,{H}_{i}^{s}+\frac{1}{RT}{\int }_{{p}_{{{\rm{H}}}_{2}{\rm{O}}}^{s}}^{p}{\bar{v}}_{i}dp$$

5 $$\begin{array}{ccc}\mathrm{ln}\,{H}_{{{\rm{CO}}}_{2}}^{s} & = & \mathrm{ln}\,{p}_{{H}_{2}O}^{s}-9.4234{({T}_{r,{{\rm{H}}}_{2}{\rm{O}}})}^{-1}+4.0087{(1-{T}_{r,{{\rm{H}}}_{2}{\rm{O}}})}^{0.355}{({T}_{r,{{\rm{H}}}_{2}{\rm{O}}})}^{-1}\\  &  & +10.3199[\exp (1-{T}_{r,{{\rm{H}}}_{2}{\rm{O}}})]{({T}_{r,{{\rm{H}}}_{2}{\rm{O}}})}^{-0.41}\end{array}$$

6 $$\begin{array}{ccc}\mathrm{ln}\,{H}_{{{\rm{CH}}}_{4}}^{s} & = & \mathrm{ln}\,{p}_{{H}_{2}O}^{s}-11.0094{({T}_{r,{{\rm{H}}}_{2}{\rm{O}}})}^{-1}+4.8362{(1-{T}_{r,{{\rm{H}}}_{2}{\rm{O}}})}^{0.355}{({T}_{r,{{\rm{H}}}_{2}{\rm{O}}})}^{-1}\\  &  & +12.5220[\exp (1-{T}_{r,{{\rm{H}}}_{2}{\rm{O}}})]{({T}_{r,{{\rm{H}}}_{2}{\rm{O}}})}^{-0.41}\end{array}$$

7 $$\mathrm{ln}(\frac{{H}_{salt,i}}{{H}_{i}})={k}_{salt,i}{m}_{salt}$$

8 $${k}_{{{\rm{salt}},{\rm{CO}}}_{2}}=0.11572-6.0293\times {10}^{-4}\hat{T}+3.5817\times {10}^{-6}{\hat{T}}^{2}-3.772\times {10}^{-9}{\hat{T}}^{3}$$

9 $$\begin{array}{rcl}{k}_{{{\rm{salt}},{\rm{CH}}}_{4}} & = & 3.38828-0.0318765T+1.22003\times {10}^{-4}{T}^{2}\\  &  & -2.31891\times {10}^{-7}{T}^{3}+2.22938\times {10}^{-10}{T}^{4}-8.83764\times {10}^{-14}{T}^{5}\end{array}$$

where *f*_*i,j*_ indicates the fugacity of species *i*, i.e., CO_2_ and CH_4_, in phase *j*; *H*_*i*_ is Henry’s law constant for species *i*; *x*_*i*_ is the mole fraction of species *i* in the aqueous phase; ${H}_{i}^{s}$ is the Henry’s law constant at the saturation pressure of H_2_O, temperature, and zero salinity; ${p}_{{{\rm{H}}}_{2}{\rm{O}}}^{s}$ is the saturation pressure of H_2_O; ${\bar{v}}_{i}$ is the partial molar volume of species *i*; ${T}_{r,{{\rm{H}}}_{2}{\rm{O}}}$ is the reduced temperature of H_2_O; ${H}_{salt,i}$ is the Henry’s law constant at the specific salinity condition; ${k}_{salt,i}$ is the salting-out coefficient; *m*_*salt*_ is the molaltiy of the dissolved salt; and $\hat{T}$ and *T* are the temperatures with °C and K.

### Geochemical reactions

During CO_2_ injection, a fraction of dissolved CO_2_ in water may react with water. It would produce H^+^ and lower pH via aqueous reactions. Because the Eagle Ford reservoir has a high content of carbonate minerals, significant dissolution of carbonate minerals may occur at low-pH conditions. Therefore, it is important to consider the major geochemical reactions, including the aqueous and mineral reactions. The mathematical formulations and database of the geochemical reactions are referred from the works^36–39^. The aqueous reaction is a homogeneous reaction, thus implying that the reactions occur only in the aqueous phase. In aqueous reactions, ions may either form complexes with other ions, or aqueous complexes may decompose to form ions. Because the aqueous reaction is fast, it obeys the law of mass action introducing the ion activity product (Eqs. 10 and 11). The acitivity of ion, i.e., an effective concentration of ion, is defined as a function of acitivity coefficient and molality of ion (Eq. 12). The B-dot model is employed to calculate the acitivity coefficient incorporating ionic strength and charge of the ion (Eq. 13).

10 $${Q}_{\alpha }-{K}_{eq,\alpha }=0\,{\rm{with}}\,\alpha =1,\ldots .\,,\,{R}_{aq}$$

11 $${Q}_{\alpha }=\mathop{\prod }\limits_{i=1}^{{n}_{aq}}{a}_{i}^{{v}_{i\alpha }},\,{\rm{with}}\,{n}_{aq}={n}_{c}+{n}_{a}$$

12 $${a}_{i}={\gamma }_{i}{m}_{i},\,{\rm{with}}\,i=1,\ldots ,{n}_{aq}$$

13 $$\log \,{\gamma }_{i}=-\frac{{A}_{\gamma }{z}_{i}^{2}\sqrt{I}}{1+{\dot{a}}_{i}{B}_{\gamma }\sqrt{I}}+\dot{B}I$$

where $\alpha$ denotes aqueous reaction; ${K}_{eq,\alpha }$ is the equilibrium constant of aqueous reaction; ${Q}_{\alpha }$ is the ion activity product of aqueous reaction; ${R}_{aq}$ is the number of reactions between components in aqueous phase; *i* indicates the component of aqueous reaction; ${n}_{aq}$ is the total number of components in the aqueous phase; ${n}_{c}$ is the number of gaseous components that are soluble in the aqueous phase; ${n}_{a}$ is the aqueous components that exist only in the aqueous phase; *a*_*i*_ is the activity; ${\gamma }_{i}$ is the ionic activity coefficient; *m*_*i*_ is the molality; *I* is the ionic strength; ${z}_{i}$ is the charge of the ion; ${\dot{a}}_{i}$ is the ion size parameter; and ${A}_{\gamma }$, ${B}_{\gamma }$, and $\dot{B}$ are the temperature-dependent parameters.

The mineral reaction of dissolution or precipitation is a heterogeneous reaction involving multiple phases, i.e., the solid and aqueous phases. Because the mineral reaction is a slow kinetic reaction, it requires enough time to achieve the equilibrium state in accordance with the rate law (Eqs. 14 and 15).

14 $${\gamma }_{\beta }={\hat{A}}_{\beta }{k}_{\beta }(1-\frac{{Q}_{\beta }}{{K}_{eq,\beta }}),\,{\rm{w}}{\rm{i}}{\rm{t}}{\rm{h}}\,\beta =1,\cdot \,\cdot \,\cdot \,,{R}_{mn}$$

15 $${Q}_{\beta }=\mathop{\prod }\limits_{k=1}^{{n}_{\beta }}{a}_{k}^{{v}_{k,\beta }}$$

where *β* denotes mineral reaction; ${r}_{\beta }$ is the mineral reaction rate; ${k}_{\beta }$ is the reaction rate constant of mineral reaction; ${\hat{A}}_{\beta }$ is the reactive surface area of a mineral; ${K}_{eq,\beta }$ is the solubility product constant of the mineral reaction; *Q*_*β*_ is the ion activity product of the mineral reaction; *R*_*mn*_ is the number of reactions between minerals and aqueous components; *k* indicates the component in mineral reaction; ${n}_{\beta }$ is the number of mineral components; ${a}_{k}$ is the activity of component *k*; and ${v}_{k\beta }$ is the stoichiometric coefficient of the mineral reaction.

The mineral reaction generates or consumes the aqueous species in water. The formation/consumption rate of the aqueous species depends on the mineral reaction rate (Eq. 16). In addition, the mineral dissolution or precipitation changes the pore volume of the reservoir. The change in the total moles of the mineral results in the change of the pore volume, i.e., porosity (Eq. 17). The increasing or decreasing porosity also affects the permeability of the reservoir (Eq. 18).

16 $${\gamma }_{k,\beta }={v}_{k,\beta }{\gamma }_{\beta }$$

17 $$\phi ={\phi }^{0}-\mathop{\sum }\limits_{\beta =1}^{{n}_{\beta }}(\frac{{N}_{\beta }}{{\rho }_{\beta }}-\frac{{N}_{\beta }^{0}}{{\rho }_{\beta }})$$

18 $$\frac{k}{{k}^{0}}={(\frac{\phi }{{\phi }^{0}})}^{3}{(\frac{1-{\phi }^{0}}{1-\phi })}^{2}$$

where ${v}_{k,\beta }$ is the stoichiometric coefficient of the mineral reaction; ${\gamma }_{k,\beta }$ is the consumption or production rate of ionic species in brine owing to the mineral reaction; *ϕ*^0^ and *ϕ* are the porosities before and after mineral reaction; ${N}_{\beta }^{0}$ and *N*_*β*_ are the total moles of mineral per bulk volume before and after mineral reaction; *ρ*_*β*_ is the mineral molar density; and *k*^0^ and *k* are the permeabilities before and after mineral reaction.

## Numerical Simulations

**Figure 1:**
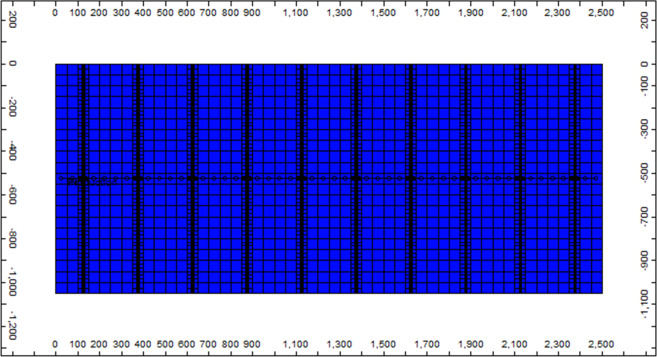
Schematic of the shale gas reservoir and its dimensions.

This study uses the GEM^TM^ software, developed by CMG Ltd, to simulate multi-phase and multi-component flows coupled with aqueous solubility and geochemical reactions. The target reservoir is constructed based on the published studies of the Eagle Ford shale gas reservoir^11,40–42^. A description of the reservoir is represented in Fig. 1. The reservoir’s dimensions are 2,500 × 1,050 × 200 ft^3^. It is discretized with 50 × 11 × 1 grid blocks. The horizontal well and hydraulic fracturing technologies are simulated in the reservoir. A total of 10 sets of transverse fractures are induced in the reservoir along with the horizontal well. This study simulates only one stage of the hydraulically fractured stimulated reservoir based on symmetry to save computation time and storage. The properties of the system are described in Table 1. The size of the fractured width is assumed to be equal to 0.001 ft referring the Rubin’s work^41^. The extended Langmuir adsorption model is used to describe the adsorptions of CH_4_ and CO_2_ on the rock surface. The parameters of the model are listed in Table 2^43^.

**Table 1:** Reservoir properties.

Properties	Values
Depth	8,608 ft
Reservoir pressure	6,568 psia
Reservoir temperature	242°F
Matrix permeability	1 × 10^−4^ md
Fracture permeability	8 × 10^−2^ md
Matrix porosity	6 × 10^−2^
Fracture porosity	1 × 10^−5^
Hydraulic fracture at half-length	500 ft
Hydraulic fracture spacing	250 ft
Hydraulic fracture conductivity	83.3 md·ft

**Table 2:** Parameters of adsorptions of CH_4_ and CO_2_.

Components	Parameters	Values
CH_4_	Langmuir pressure (*p*_*L*_)	694.7 psia
	Langmuir volume (*V*_*L*_)	12.7 scf/ton
CO_2_	Langmuir pressure (*p*_*L*_)	409.6 psia
	Langmuir volume (*V*_*L*_)	33.1 scf/ton

The CO_2_ huff-n-puff process is adopted as the CO_2_ injection to enhance natural gas production from shale reservoir. Prior to the execution of the CO_2_ huff-n-puff process, natural depletion process recovers the gas over a two-year period. When the gas production by natural depletion becomes negligible, the CO_2_ huff-n-puff process is deployed for the next two-year period. The bottom-hole pressure of the producer is set to 1,875 psi. During the CO_2_ huff-n-puff, the CO_2_ is injected with 2 MMscf/day until the bottom-hole pressure of the injector reaches 9,000 psi. A total of 12 cycles of CO_2_ huff-n-puff are designed. Each cycle comprises two processes for two months—CO_2_ injection for one month and production for another month.

## Results

This study consists of two sections: (1) simulations incorporating aqueous solubilities of CO_2_ and CH_4_ and (2) simulations coupled with geochemistry. The first section of the simulations investigates the effect of aqueous solubility on the hydrocarbon production and CO_2_ sequestration during the CO_2_ huff-n-puff process. The second section quantifies the effect of the geochemical reactions—which is attributed to the CO_2_ dissolution in water—on the performance of the CO_2_ huff-n-puff process in the shale gas reservoir.

### CO_2_ huff-n-puff process and consideration of the aqueous solubility

**Figure 2:**
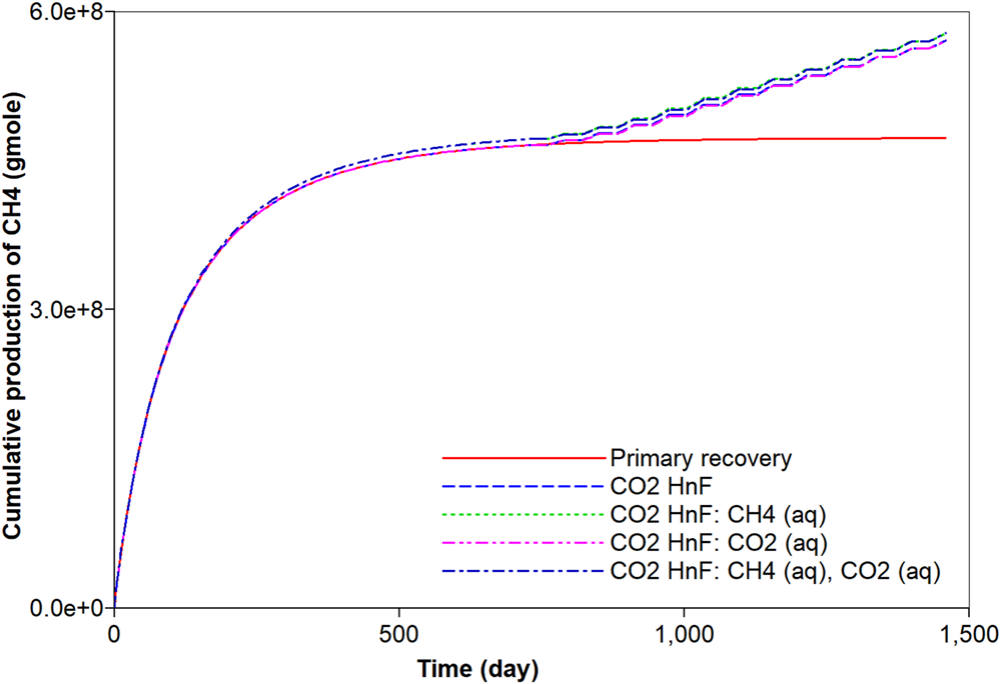
Effects of aqueous solubility of CO_2_ and CH_4_ on cumulative gas production (mole) during primary recovery and CO_2_ huff-n-puff processes.

**Figure 3:**
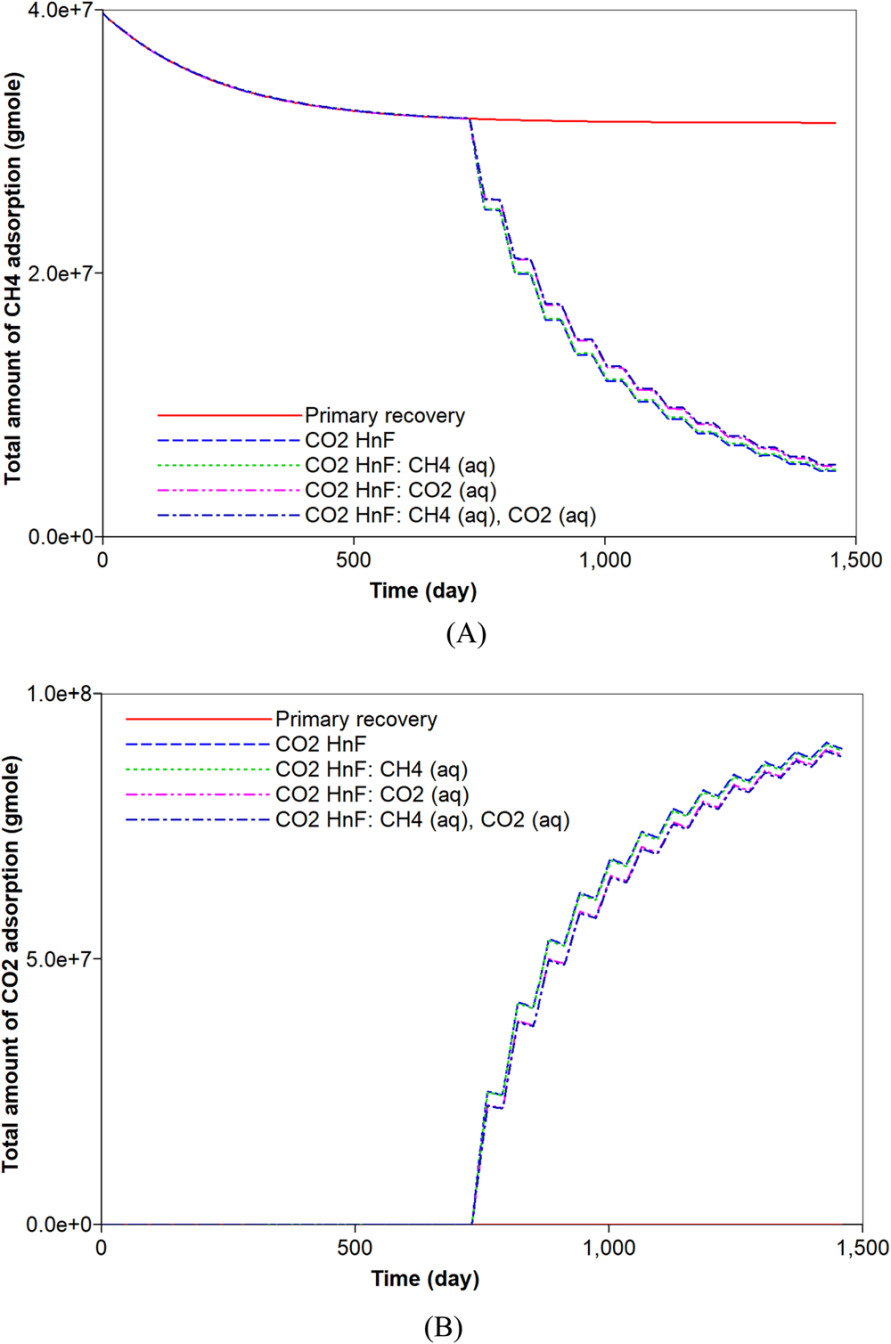
Effects of aqueous solubility of CO_2_ and CH_4_ on the adsorption (moles) of (**A**) CH_4_ and (**B**) CO_2_ during primary recovery and CO_2_ huff-n-puff processes.

**Figure 4:**
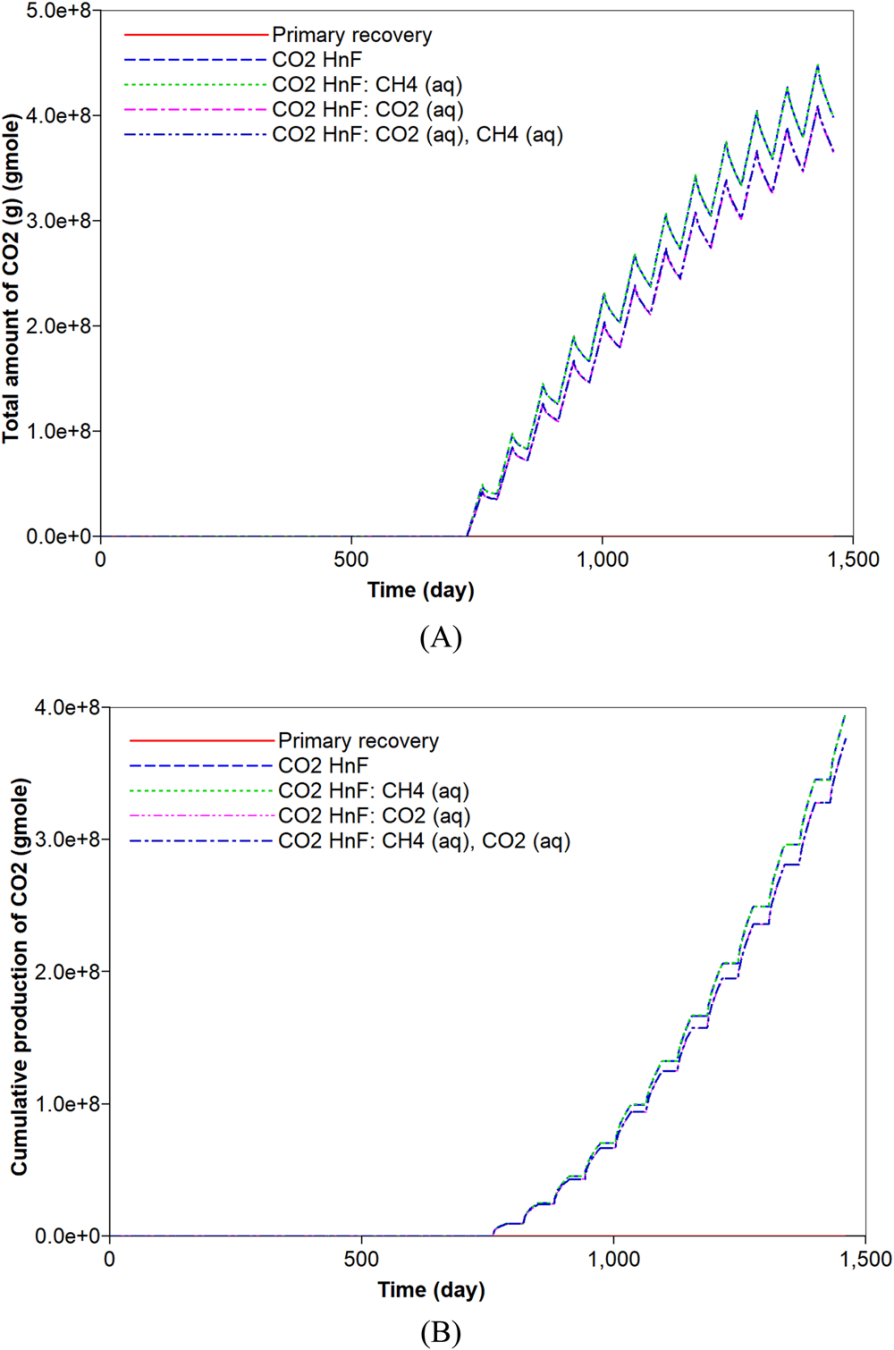
Effects of aqueous solubility of CO_2_ and CH_4_ on (**A**) CO_2_ storage by free gas (moles) in a shale reservoir and (**B**) cumulative production (moles) of CO_2_ during primary recovery and CO_2_ huff-n-puff processes.

The original gas in place (OGIP) is initially determined to be equal to 6.7 × 10^8^ moles. This corresponds to 563.5 MMscf of which free and adsorbed gases respectively occupy 94.1% and 5.9% of OGIP. For a comparison to the simulation of the CO_2_ huff-n-puff process, the primary recovery process of natural depletion is simulated for a period of four years. Figure 2 describes the cumulative gas production of CH_4_ from the shale reservoir. The primary recovery process recovers 70% of OGIP. During the pressure depletion, 21% of adsorbed CH_4_ is produced and it corresponds to 1.7% of the total CH_4_ production (Figs. 2 and 3A). Most of the gas production is attributed to the production of free CH_4_ gas. Because the free gas production is relatively fast, the gas production rate significantly decreases after one year. The rapid decline in the early production initiates the CO_2_ huff-n-puff process. Firstly, the simulation of the CO_2_ huff-n-puff process neglects the aqueous solubility and it is the base case of the CO_2_ huff-n-puff process. The CO_2_ injection would pressurize the depleted reservoir and cause more desorption of CH_4_ due to the increasing adsorption of CO_2_ (Fig. 3). The CO_2_ huff-n-puff process injects 734.5 MMscf of CO_2_ (or equivalently 8.8 × 10^8^ mol), and 10.1% of the injected CO_2_ is adsorbed on the rock surface (Fig. 3B). As a result, the CO_2_ huff-n-puff process produces a total 84.7% of OGIP, which corresponds to a 14.7% higher production of OGIP than the primary recovery process for four years (Fig. 2). The CO_2_ huff-n-puff process introduces two effects of more CH_4_ desorption and re-pressurization, thereby enhancing gas recovery over primary recovery process. The CH_4_ desorption caused by CO_2_ adsorption is responsible for 5.2% of the OGIP, and corresponds to 87.4% of the initial amount of the adsorbed CH_4_ (Fig. 3A). Re-pressurization by CO_2_ injection contributes enhanced gas production as much as 9.5% of the OGIP. The CO_2_ huff-n-puff process is also effective in storing CO_2_ in the depleted reservoirs. This simulation observes that 45.1% and 10.1% of injected CO_2_ are sequestrated by geological and adsorption trappings, respectively (Figs. 3B and 4).

**Figure 5:**
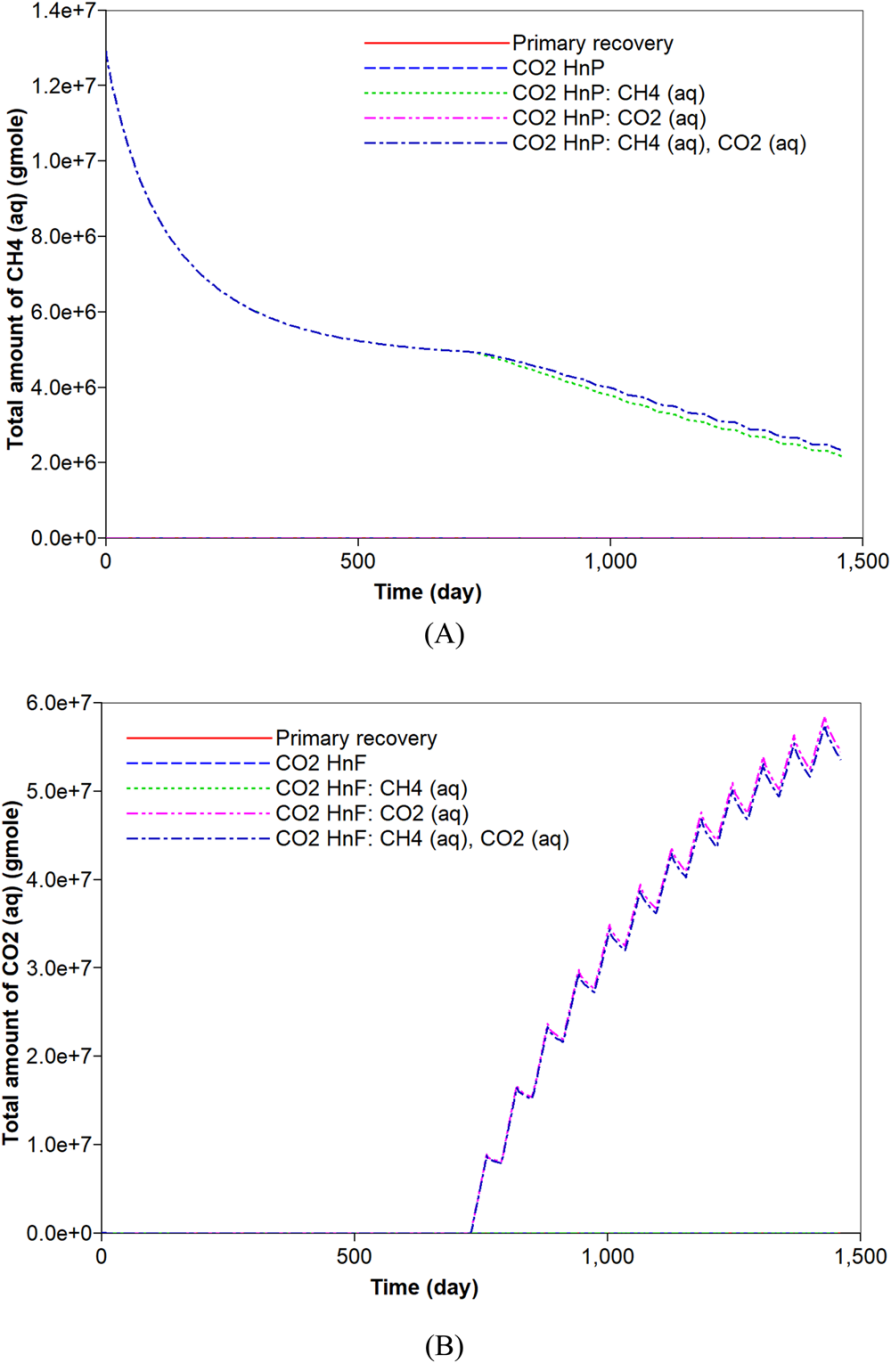
Effects of aqueous solubility of CO_2_ and CH_4_ on the dissolved (**A**) CH_4_ (moles) and (**B**) CO_2_ (moles) in water during primary recovery and CO_2_ huff-n-puff processes.

Generally, CH_4_ can dissolve in water owing to high pressure condition. Because high-pressure conditions exist within the reservoir, ignoring the aqueous solubility of CH_4_ leads to the underestimation of the initial OGIP in shale formations. When the initial gas storage process takes into consideration the mechanism of the aqueous solubility of CH_4_, the initial OGIP slightly increases by 1.9%. Because the dissolved CH_4_ in water is gasified by pressure depletion, it can be recovered (Fig. 5A). The CO_2_ huff-n-puff process is simulated in the system. The desorption of CH_4_ does not change, regardless of the aqueous solubility of CH_4_ (Fig. 3A). As a result, the CO_2_ huff-n-puff process that has accounted for the aqueous solubility of CH_4_ recovers 1.3% more CH_4_ compared to the base case (Fig. 2). The increase in the production is fully attributed to an increase in the OGIP owing to the aqueous solubility of CH_4_ (Fig. 5A). In addition, the aqueous solubility of CH_4_ hardly influence the potential of CO_2_ sequestration compared to the base case (Fig. 4B).

CO_2_ is also soluble in water. Its solubility is higher than that of CH_4_^44^. When the aqueous solubility of CO_2_ is accounted for in the simulation of the CO_2_ huff-n-puff process, there is a negligible change in the total hydrocarbon production compared to the base case (Fig. 2). However, the capacity of CO_2_ storage would be increased, and the less production of CO_2_ is observed (Fig. 4B). In this simulation, 41.2% and 10.1% of the injected CO_2_ are sequestrated in the geological and adsorption forms, respectively (Figs. 3B and 4A). In addition, 6.2% of the injected CO_2_ is captured based on an aqueous solubility mechanism (Fig. 5B) that constitutes one of the CO_2_ storage mechanisms^45^. Compared to the previous simulation that accounts for the aqueous CH_4_ solubility, the simulation that accounts for the aqueous solubility of CO_2_ leads to a smaller storage of CO_2_ via geological and adsorption trappings (Figs. 3B and 4A), but stores additional CO_2_ based on aqueous solubility (Fig. 5B). When the simulation of the CO_2_ huff-n-puff process considers the aqueous solubilities of CH_4_ and CO_2_, increasing CH_4_ production and CO_2_ sequestration are obtained comparing to the base case (Figs. 2, 3B, 4B and 5B).

### CO_2_ huff-n-puff coupling with geochemistry

Previous simulations investigate the effect of the aqueous solubility on the gas recovery and CO_2_ sequestration during the CO_2_ huff-n-puff process in the shale formation. Once the CO_2_ dissolves in brine with a decreasing pH, geochemical reactions occur in carbonate-rich shale reservoirs. This section explores the role of geochemistry on the performance of the CO_2_ huff-n-puff process in carbonate-rich shale reservoirs. Table 3 lists the geochemical reactions of the aqueous and mineral reactions for their implementations in the simulations. The reservoir is assumed to have 30% of carbonate minerals (calcite, dolomite, and magnesite) to represent a carbonate-rich shale reservoir. Simulations of the primary recovery and the CO_2_ huff-n-puff processes are conducted for the shale reservoir once the geochemical reactions have been incorporated in the simulation framework. The findings are compared to those obtained from the base case, which is the simulation of the CO_2_ huff-n-puff process based on the consideration of the aqueous solubility of CO_2_ (but not based on geochemistry). Additional application of the CO_2_ huff-n-puff process pertaining to the reservoir which has no carbonate mineral is simulated to quantify the role of the mineral. Lastly, the effect of the aqueous solubility of CH_4_ is also confirmed when the CO_2_ huff-n-puff process has accounted for geochemistry and is deployed in the carbonate-rich shale gas reservoir.

**Table 3:** Geochemical reactions.

	Reactions
Aqueous reactions	CO_2_ + H_2_O↔H^+^ + HCO_3_^−^
	H^+^ + OH^−^↔H_2_O
	H^+^ + CaOH^+^↔Ca^2+^ + HCO_3_^−^
	CaHCO_3_^+^↔Ca^2+^ + HCO_3_^−^
	CaSO_4_↔Ca^2+^ + SO_4_^2−^
Mineral reaction	Calcite + H^+^↔Ca^2+^ + HCO_3_^−^
	Dolomite + H^+^↔Ca^2+^ + Mg^2+^ + 2HCO_3_^−^
	Magnesite + H^+^↔Mg^2+^ + HCO_3_^−^

**Figure 6:**
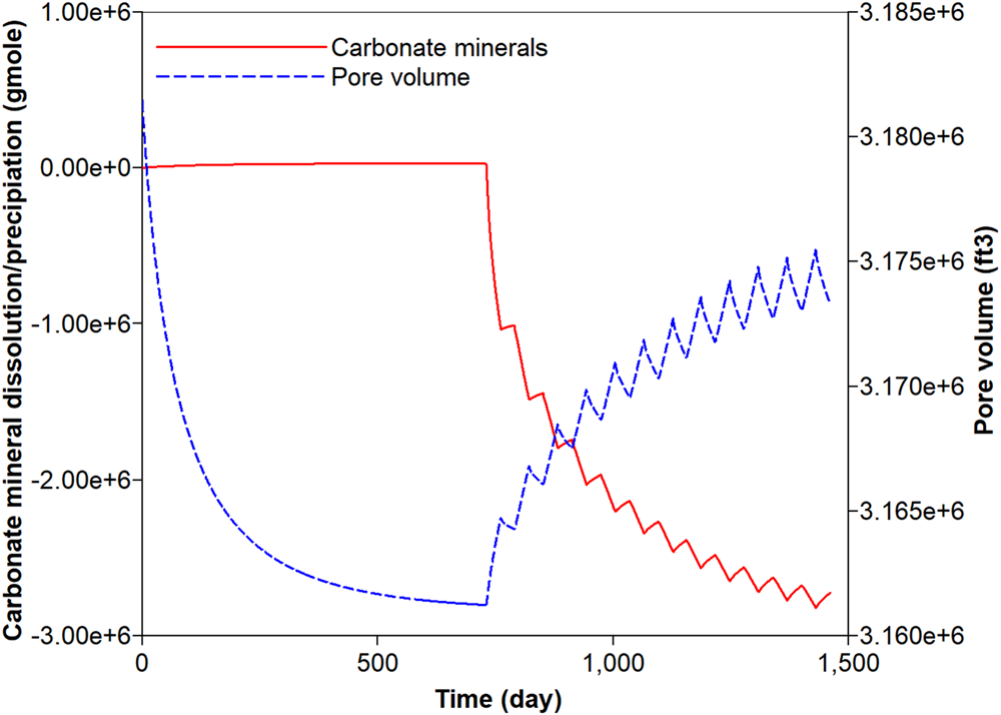
Dissolution/precipitation of carbonate minerals (moles) and pore volume (ft^3^) of shale reservoir during the CO_2_ huff-n-puff process considering geochemistry.

**Figure 7:**
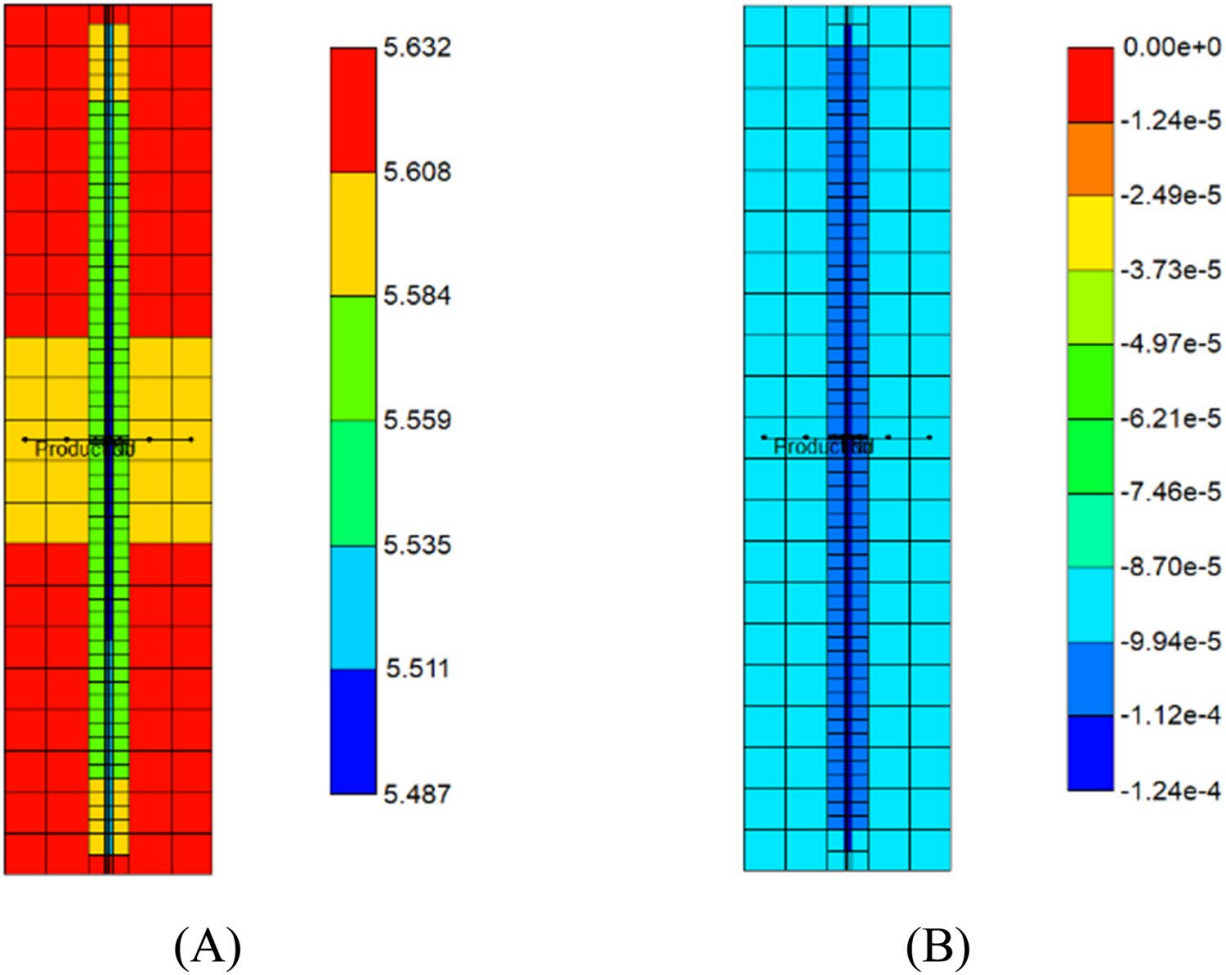
Distribution of (**A**) pH and (**B**) porosity change in the shale reservoir after the CO_2_ huff-n-puff process considering geochemistry.

**Figure 8:**
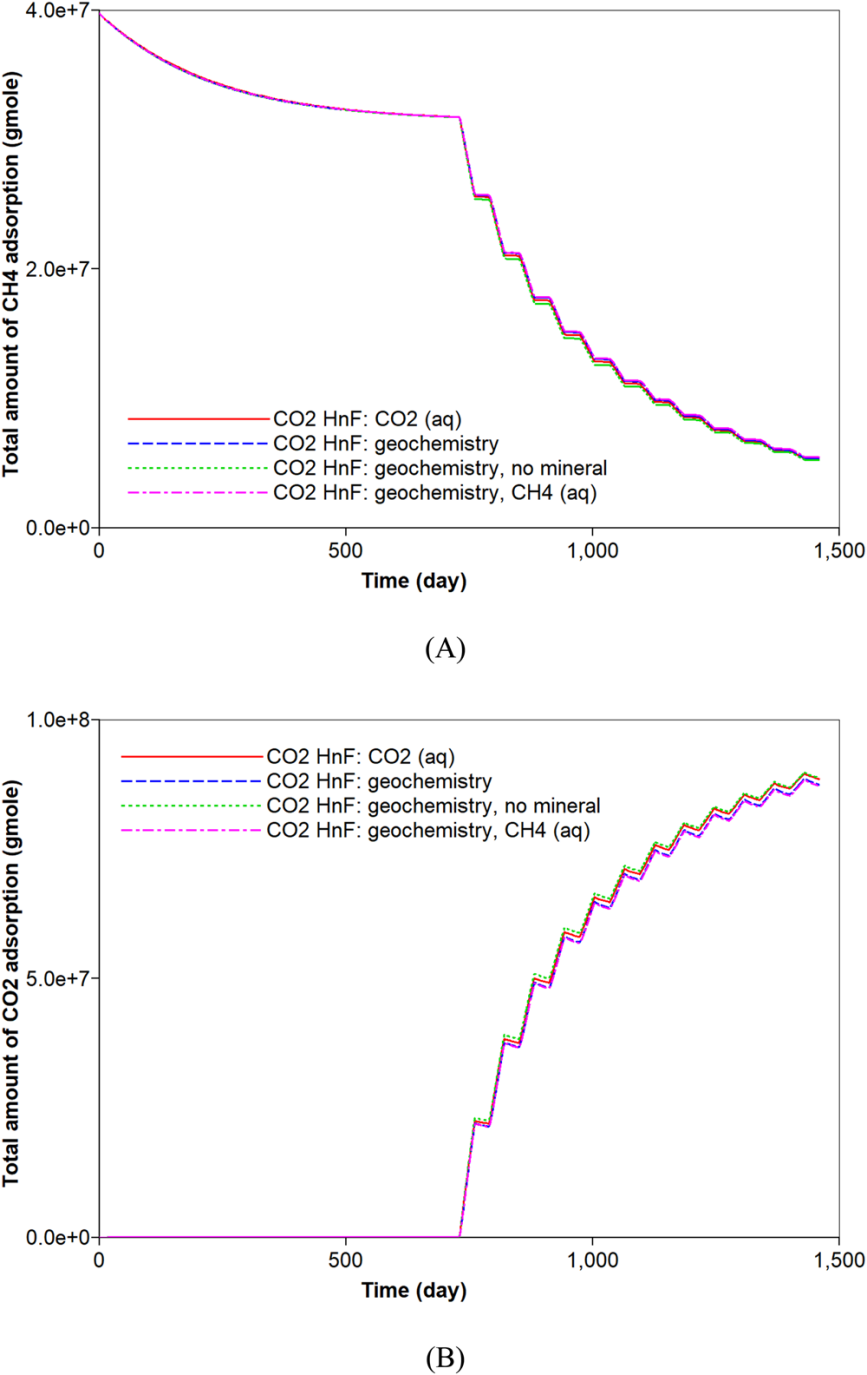
Effects of aqueous solubility and geochemistry on the adsorption (moles) of (**A**) CH_4_ and (**B**) CO_2_ during CO_2_ huff-n-puff processes.

**Figure 9:**
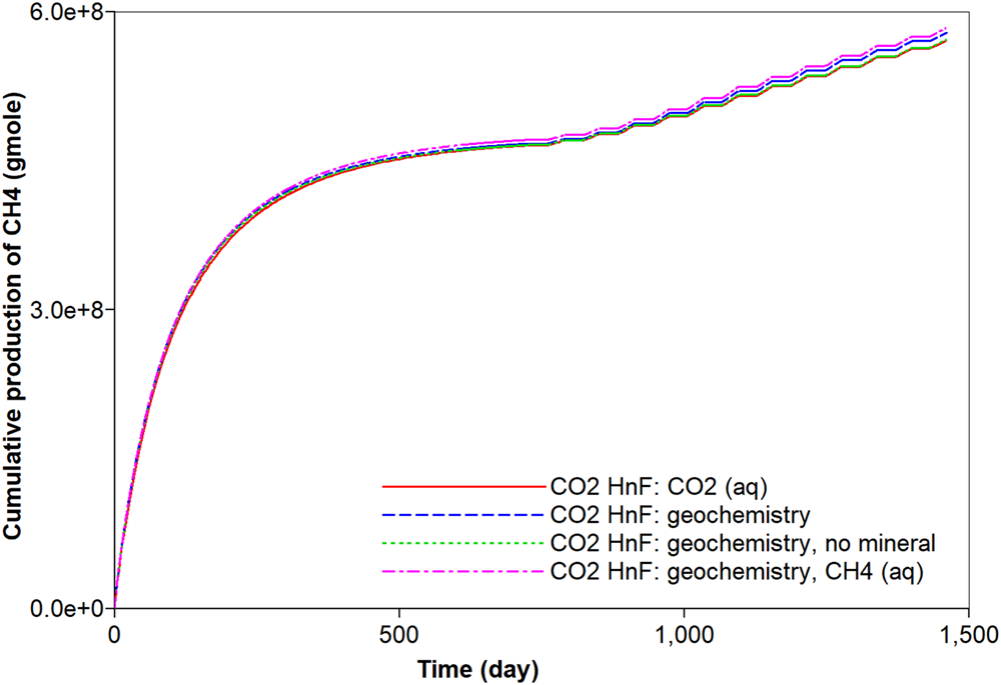
Effects of aqueous solubility and geochemistry on cumulative gas production (moles) of CH_4_ during CO_2_ huff-n-puff processes.

First, the simulation results of the carbonate-rich shale reservoir are analyzed. Prior to the CO_2_ huff-n-puff process, the precipitation of carbonate minerals mainly occurs during the natural depletion process (Fig. 6). The precipitation of carbonate minerals slightly decreases the pore volume of the reservoir, but the effect of the rock’s compressibility owing to the pressure depletion overwhelmingly decreases the pore volume (Fig. 6). Once the CO_2_ huff-n-puff process is initiated, the dissolution of CO_2_ in water produces H^+^ and decreases pH (Fig. 7A). In low-pH conditions, carbonate minerals dissolve. The mineral dissolution leads to an increase in the pore volume (Fig. 6). During the CO_2_ huff-n-puff process, the pore volume of the reservoir is still lower than initial volume despite of re-pressurization. The dissolution of carbonate minerals enlarges the pore volume, i.e., the porosity (Fig. 7B). This is equivalent to a minor increase in the permeability of the matrix. The behaviors of adsorption and desorption of CH_4_ and CO_2_ hardly change regardless of geochemistry (Fig. 8). As a result, the simulation of the CO_2_ huff-n-puff process incorporating geochemistry recovers additional amount of CH_4_ by 1.7% compared to the base case (Fig. 9). The increase in the gas production is attributed to the dissolution of carbonate minerals enlarging the porosity during the CO_2_ huff-n-puff process. This can be confirmed by comparing these findings to the simulation findings of the CO_2_ huff-n-puff process for the reservoir which contains no carbonate minerals. When the process is deployed into the reservoir, any increase in the cumulative production is not observed (Fig. 9). When the CO_2_ huff-n-puff process incorporating the aqueous solubility of CH_4_ as well as geochemistry is simulated in the carbonate-rich shale formation, the cumulative production of CH_4_ is increased by 2.6% compared to the base case (Fig. 9). Because the geochemical reactions and aqueous solubility of CH_4_ negligibly affect the adsorption and desorption of CO_2_ and CH_4_ (Fig. 8), an increase in hydrocarbon production is attributed to an increase in the initial OGIP owing to the solubilized CH_4_ in water, and to the porosity changes owing to dissolution of carbonate minerals.

**Figure 10:**
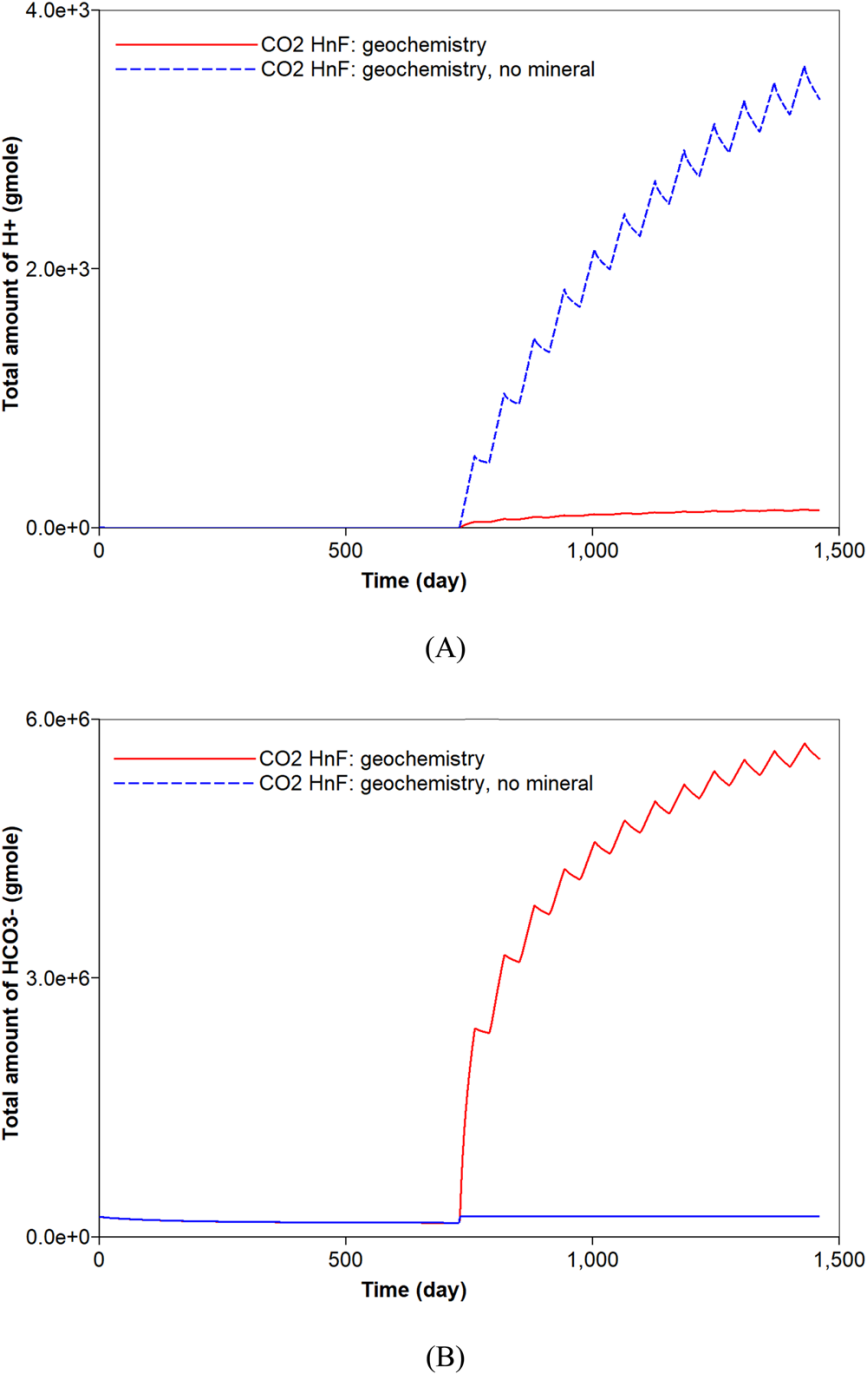
Effect of mineral reactions on the total amount (moles) of (**A**) H^+^ and (**B**) HCO_3_^−^ in the shale reservoir during CO_2_ huff-n-puff processes considering geochemistry.

**Figure 11:**
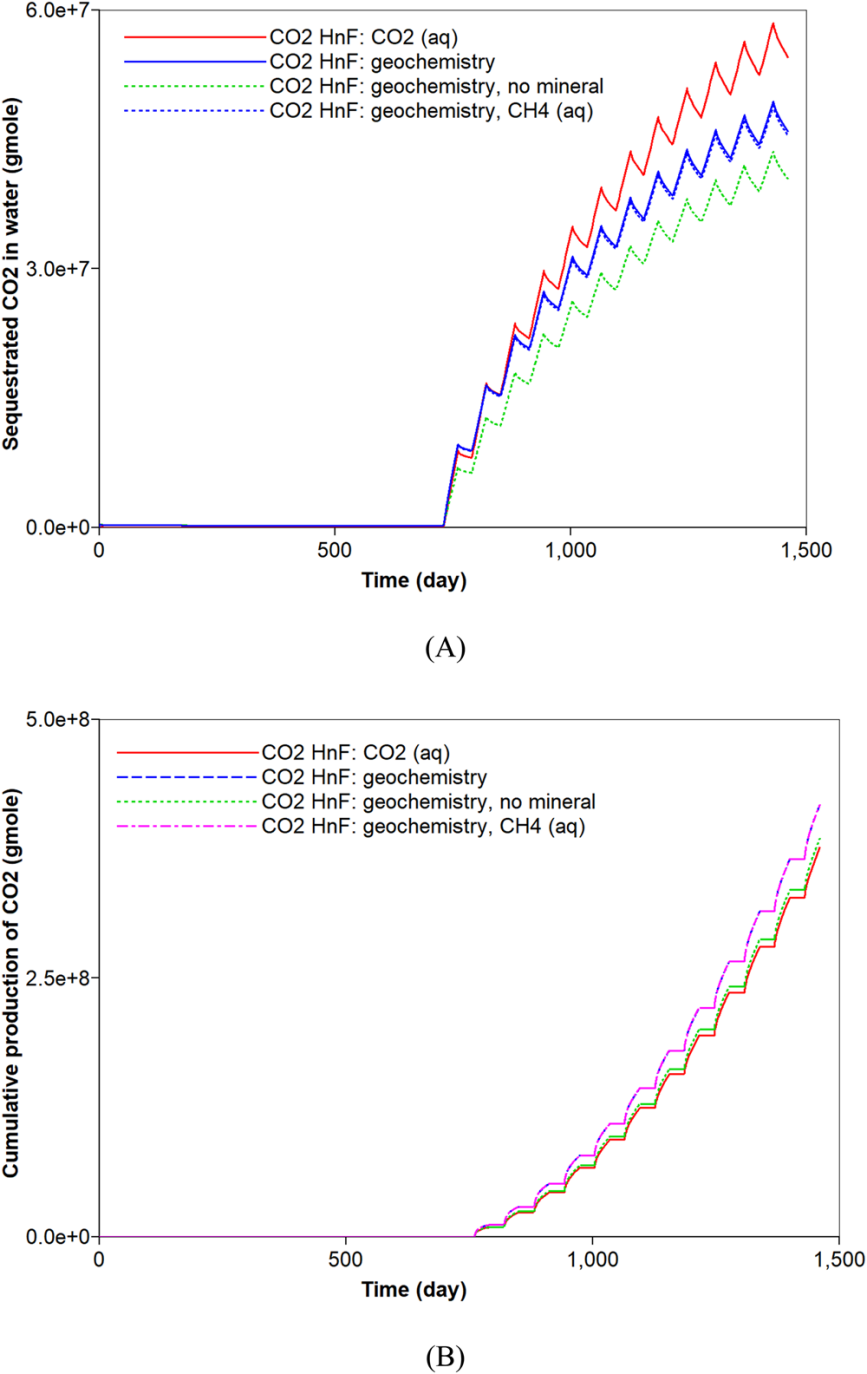
Effects of aqueous solubility and geochemistry on the (**A**) dissolved CO_2_ (moles) in water and (**B**) cumulative production (moles) of CO_2_ during CO_2_ huff-n-puff processes.

The mineral dissolution influences the concentration of ions in water. It produces HCO_3_^−^ in water buffering pH of *in-situ* brine. When the reservoir has no carbonate minerals, higher concentrations of H^+^ and lower concentrations of HCO_3_^−^ are observed during the CO_2_ huff-n-puff process (Fig. 10). The geochemical reactions also affect the CO_2_ storage capacity. Precipitation of carbonate minerals contributes to the long-term CO_2_ sequestration that constitutes one of the CO_2_ storage mechanisms^45^. The dissolution of carbonate minerals observed in the CO_2_ huff-n-puff process is unfavorable to the CO_2_ storage. In addition, the dissolved CO_2_ in water via solubility trapping mechanism can exist in different forms of carbon dioxide complexes, e.g., HCO_3_^−^, CaHCO_3_^+^. As a result, the CO_2_ huff-n-puff process with geochemistry sequesters a smaller amount of CO_2_ in water (approximately 14.6%) compared to the base case (Fig. 11A). Because there is a negligible change in the adsorption of CO_2_ (Fig. 8B), a smaller CO_2_ storage in water mainly causes an increased CO_2_ production of up to 8.6% (Fig. 11B). When the reservoir has no carbonate minerals, the sequestered CO_2_ in water decreases by at most 13.2% compared to the simulation result with the carbonate-rich shale reservoir (Fig. 11A). A smaller production of CO_2_ (up to 6.0%) is expected when the reservoir does not contain any carbonate minerals (Fig. 11B). Lastly, simulations investigate whether the aqueous solubility of CH_4_ affects the CO_2_ storage of CO_2_ huff-n-puff process considering geochemistry or not. Because the aqueous dissolution of CH_4_ has no impact on the geochemical reactions, it hardly affects the capacity of CO_2_ sequestration (Fig. 11B).

## Conclusions

This study assessed the hydrocarbon recovery and CO_2_ storage during the CO_2_ huff-n-puff process in shale gas reservoirs. It explored the roles of aqueous solubility and geochemistry on the performance of the CO_2_ huff-n-puff process. The following conclusions have been drawn based on the findings of the numerical study.

1. While most of the OGIP in the shale gas reservoir consisted of free gas and the adsorbed CH_4_, neglecting the aqueous solubility of CH_4_ was shown to underestimate the OGIP by 1.9% and gas recovery by 1.3%. Because the dissolved CH_4_ in water can be recoverable during the natural depletion and the CO_2_ huff-n-puff process, accurate prediction of the hydrocarbon recovery required the consideration of the aqueous solubility of CH_4_.
2. Consideration of the aqueous solubility of CO_2_ did not affect the gas recovery of CH_4_ from the shale formation. However, it sequestered additional 6.1% of injected CO_2_ in the shale formation via the aqueous solubility. During the CO_2_ huff-n-puff process, accurate prediction of CO_2_ sequestration should consider storing CO_2_ via forms of geological trapping, adsorption, and solubility trapping in depleted shale formation.
3. With the incorporation of geochemistry in the simulation of the CO_2_ huff-n-puff process, the dissolution of CO_2_ promoted the recovery of CH_4_ from the carbonate-rich shale gas reservoir. Once the CO_2_ dissolved in water, the pH of brine decreased and the dissolution of carbonate minerals occurred in low-pH condition. This resulted in a slight increase in the pore volume, i.e., porosity, and in a parallel increase in permeability, and then an increase in the hydrocarbon production of the order of 2.6% was obtained. Therefore, it is necessary to consider the effect of geochemistry for accurate prediction of shale gas production of CO_2_ huff-n-puff in carbonate-rich shale formations.
4. Ignoring the geochemical reactions was associated with the risk of overestimating the CO_2_ sequestration during the CO_2_ huff-n-puff process. Because of the geochemical reactions, dissolved CO_2_ can be sequestered into the forms of carbon dioxide complexes in water. Therefore, consideration of the geochemical reaction predicted a smaller potential of CO_2_ sequestration by 14.6% during the CO_2_ huff-n-puff process in the depleted shale gas reservoir.
