# Author Correction: Effects of Aqueous Solubility and Geochemistry on CO_2_ Injection for Shale Gas Reservoirs

**DOI:** 10.1038/s41598-021-04141-7

**Published:** 2021-12-27

**Authors:** Ji Ho Lee, Jinhyung Cho, Kun Sang Lee

**Affiliations:** 1 Korea National Oil Corporation, 305, Jongga-ro, Jung-gu, Ulsan, 44538 Republic of Korea; 2 Department of Earth Resources and Environmental Engineering, Hanyang University, Seoul, 04763 Republic of Korea

Correction to: *Scientific Reports* 10.1038/s41598-020-59131-y, published online 07 February 2020

The original version of the Article contained an error in the Author Information section, where the statement of equal contribution was implemented erroneously: “These authors contributed equally: Ji Ho Lee, Jinhyung Cho and Kun Sang Lee.”. Ji Ho Lee is the first author, Jinhyung Cho is the second author and Kun Sang Lee is the corresponding author of this Article.

The original Article has been corrected.

